# Supplementary material for: The mycobacterial phosphatase PtpA regulates the expression of host genes and promotes cell proliferation
Source: Nat Commun. 2017 Aug 15;8:244. doi: 10.1038/s41467-017-00279-z (PMC5557760; doi:10.1038/s41467-017-00279-z)
Supplement: Supplementary file 1 — Supplementary Information [file 41467_2017_279_MOESM1_ESM.pdf]

File name: Supplementary Information
Description: Supplementary Figures and Supplementary Table

File name: Supplementary Data 1
Description: The list of 3351 candidate Mtb PtpA-specific ChIP-seq signals.

File name: Supplementary Data 2
Description: The list of 280 candidate Mtb PtpA-regulated protein-coding genes.

File name: Peer Review File
Description:

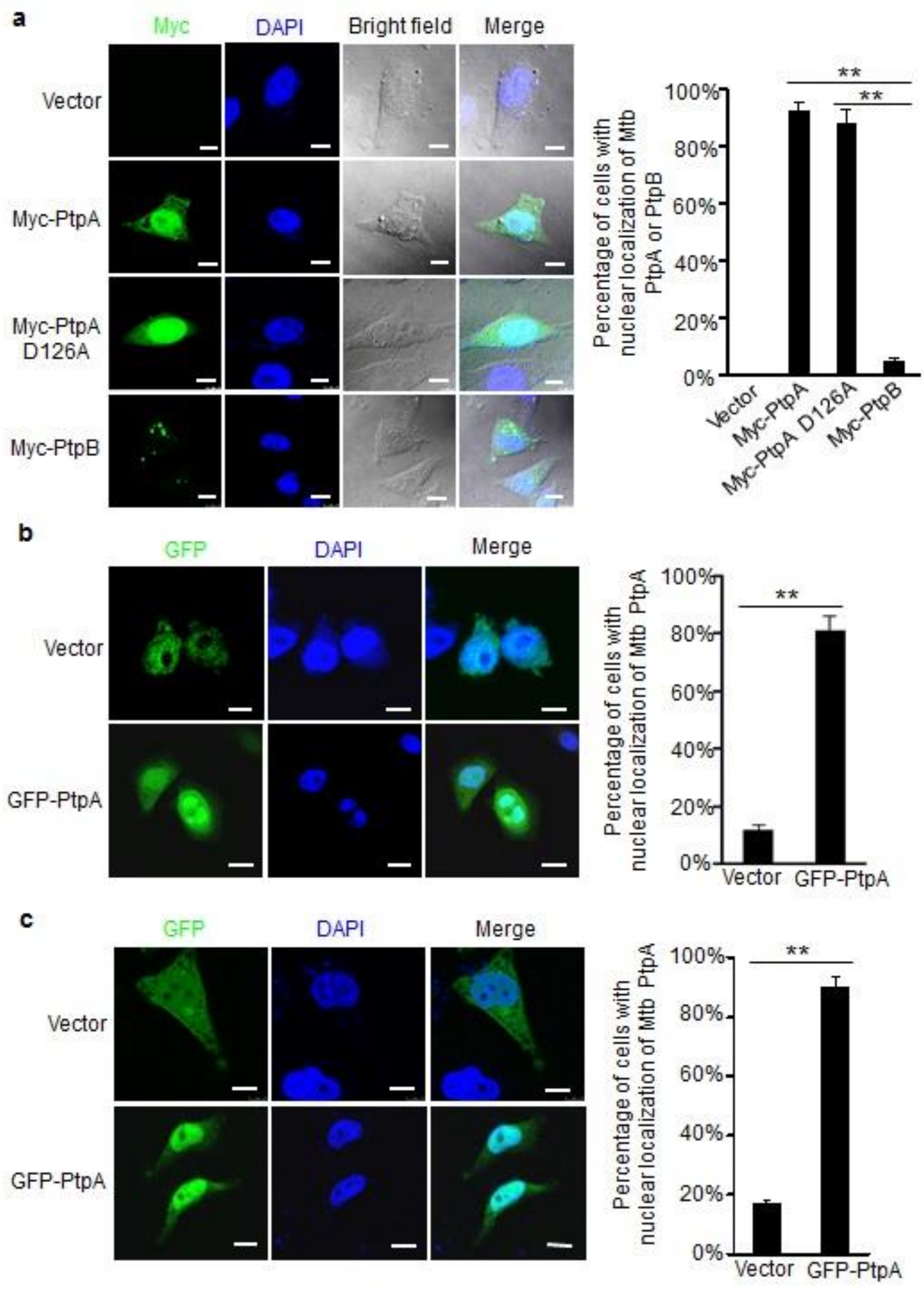


**Supplementary Figure 1: *M. tuberculosis* PtpA is present both in the cytoplasm and nucleus of host cells.** (**a**) U937 cells were transfected with an empty vector or a vector encoding Myc-tagged wild-type PtpA (Myc-PtpA, green), or PtpA D126A (Myc-PtpA D126A, green), or PtpB (Myc-PtpB, green), and then examined by confocal microscopy at 24 h post-transfection. (**b, c**) U937 cells (**b**) or A549 cells (**c**) were transfected with an empty

vector encoding GFP (green) alone or GFP-PtpA (green), and then examined by confocal microscopy at 24 h post-transfection. Nuclei (blue) were stained with DNA-binding dye (DAPI). Scale bars, 10 μm. Right, percentage of cells with nuclear localization of *M. tuberculosis* (Mtb) PtpA or PtpB. About 200 cells were counted. *$P < 0.05$ and ** $P < 0.01$ (unpaired two-tailed Student's *t* test). Data are representative of one experiment with at least three independent biological replicates (**a-c**; mean and s.e.m., n = 3).

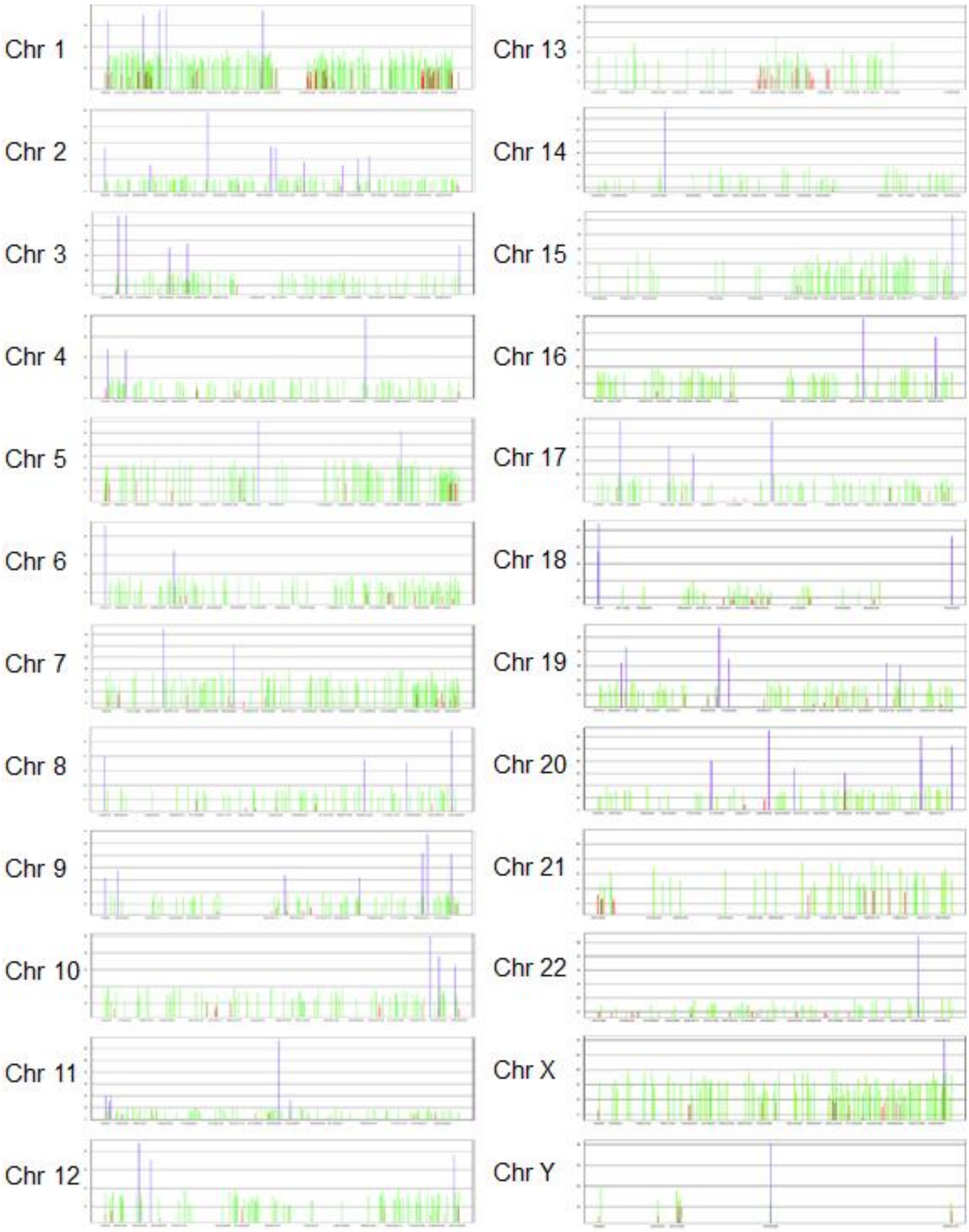


**Supplementary Figure 2: Distribution of the 3351 PtpA ChIP-seq signals in chromosomes.** Red: fold-enrichment < 10; Green: 10 < fold-enrichment < 20; Purple: fold-enrichment > 20.

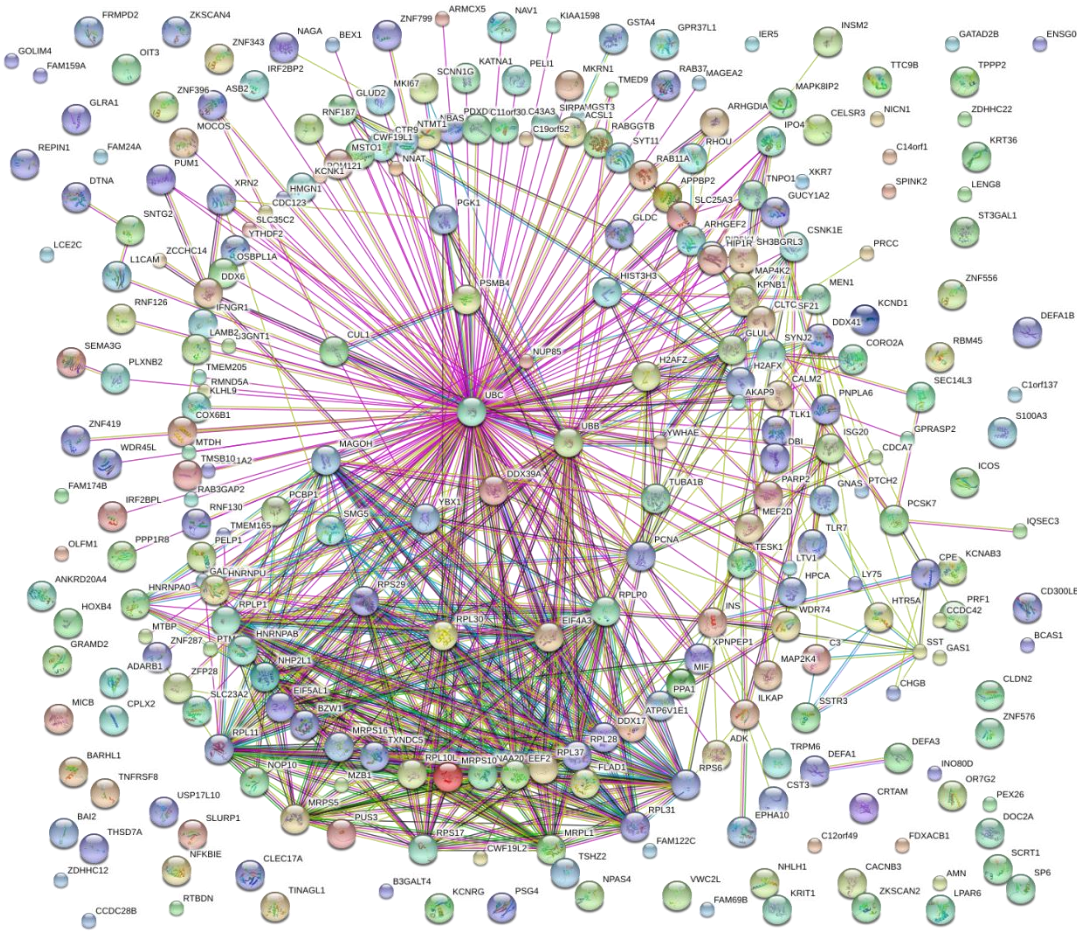


**Supplementary Figure 3: The network of proteins encoded by 280 potential PtpA target genes.** The protein-protein interaction network was visualized by the STRING 10.0.

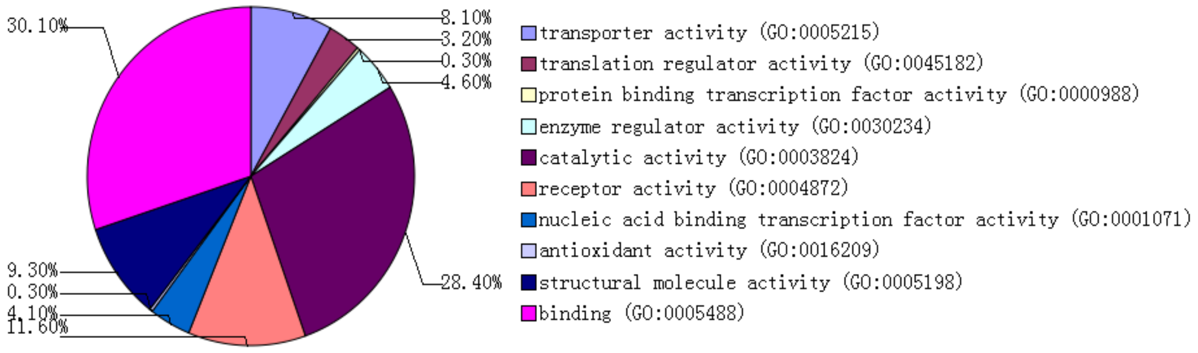


**Supplementary Figure 4: Gene ontology enrichment analysis results.** The pie chart presents the specific ontology (GO) terms for proteins encoded by 280 potential PtpA target genes.

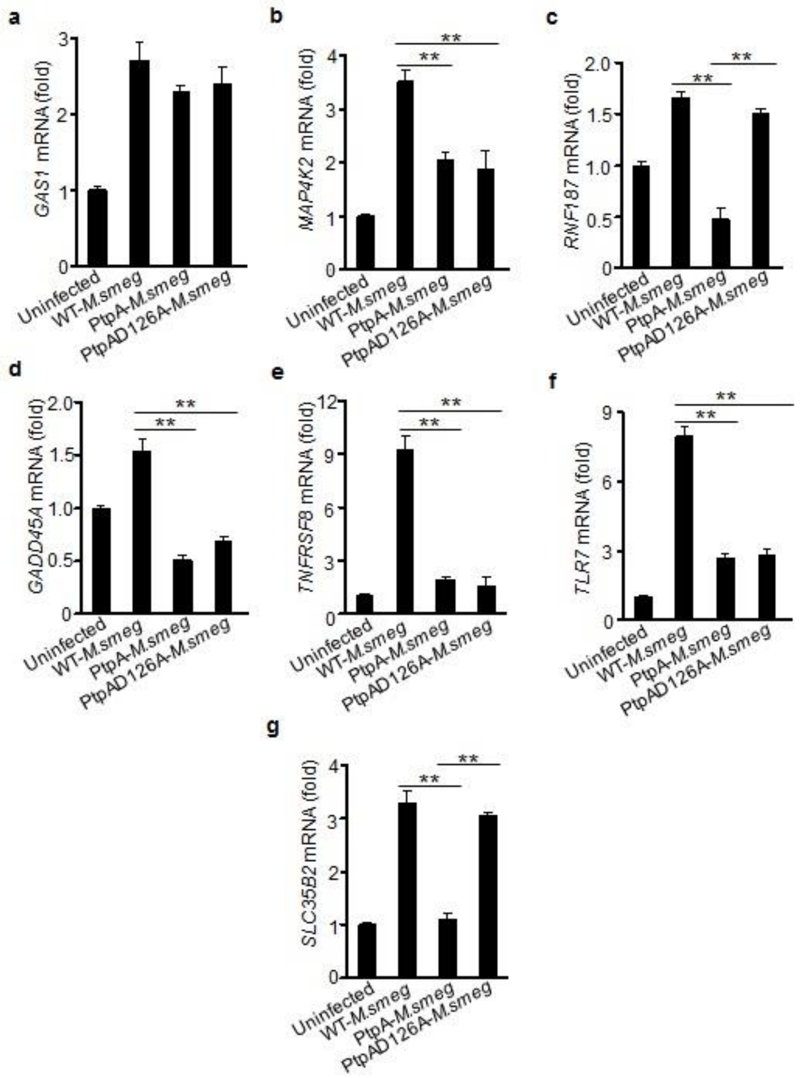


**Supplementary Figure 5: Transcriptional regulation of potential target genes by PtpA.** Quantitative PCR analysis of *GAS1*, *MAP4K2*, *RNF187*, *GADD45A*, *TNFRSF8*, *TLR7* and *SLC35B2* mRNAs were performed using U937 cells infected with wild-type *M. smegmatis* (WT-*M. smeg*), or Mtb PtpA-overexpressing *M. smegmatis* (PtpA-*M. smeg*), or PtpA D126A-overexpressing *M. smegmatis* (PtpA D126A-*M. smeg*) for 12 h. Analysis of each gene was normalized to the gene *Gapdh*. **P* < 0.05 and ** *P* < 0.01 (unpaired two-tailed Student's *t* test). Data are representative of one experiment with at least three independent biological replicates (**a-g**; mean and s.e.m., n = 3).

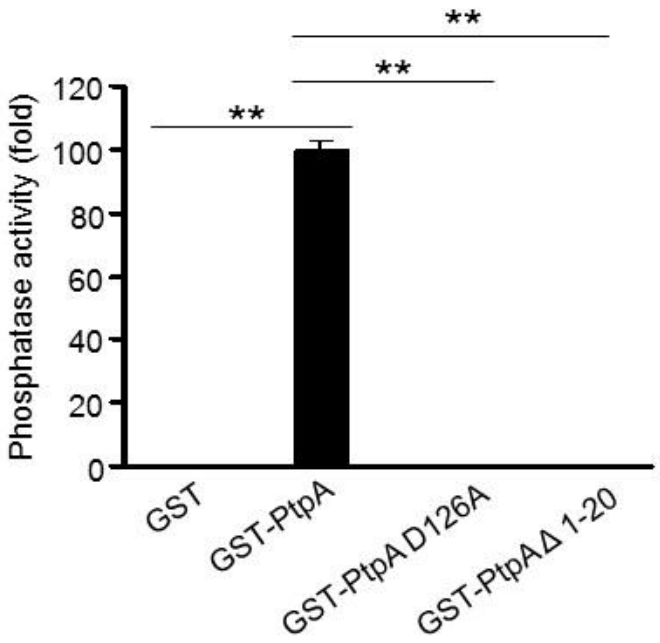


**Supplementary Figure 6: The DNA-binding region of PtpA is required for its phosphatase activity.** Para-nitrophenyl phosphate phosphatase assay. Purified GST, GST-PtpA, GST-PtpA D126A, or GST-PtpA Δ 1-20 proteins (2.5 μM) were incubated with 10 mM para-nitrophenyl phosphate (pNPP) at 37℃ for 30 min. OD value was read at 405 nm. *$P < 0.05$ and ** $P < 0.01$ (unpaired two-tailed Student's *t* test). Data are representative of one experiment with at least three independent biological replicates (mean and s.e.m., n = 3).

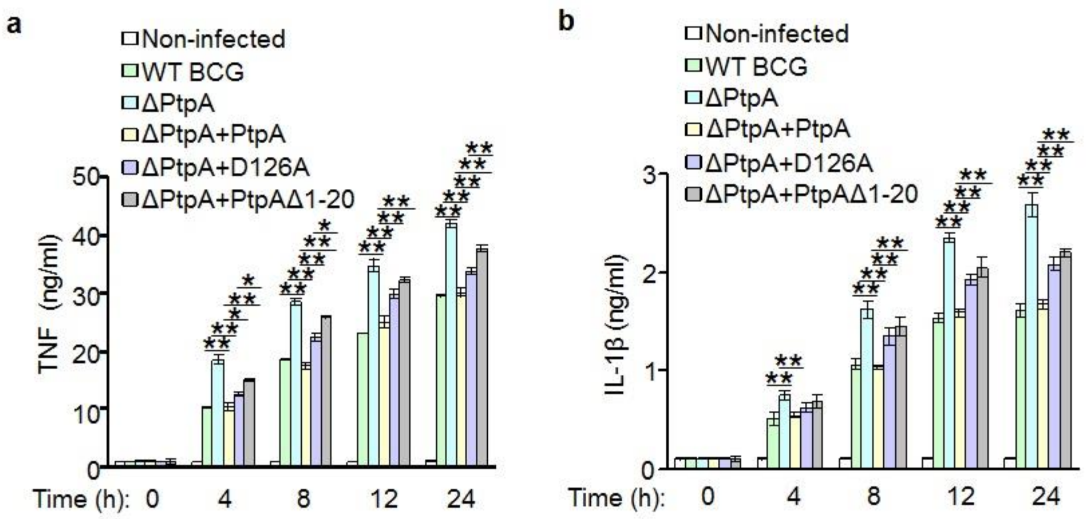


**Supplementary Figure 7: Deletion of the DNA-binding region of PtpA abolishes its inhibitory effects on the production of TNF and IL-1β.** Enzyme-linked immunosorbent assay (ELISA) of TNF (**a**) and IL-1β (**b**) in the medium of U937 cells infected with WT BCG, or PtpA-deleted BCG (BCG ΔPtpA), or BCG ΔPtpA complemented with WT PtpA (ΔPtpA + PtpA), or BCG ΔPtpA complemented with PtpA D126A (ΔPtpA + D126A), or BCG ΔPtpA complemented with PtpA Δ1-20 (ΔPtpA + PtpA Δ1-20) at a MOI of 10. **P* < 0.05 and ** *P* < 0.01 (unpaired two-tailed Student's *t* test). Data are representative of one experiment with at lease three independent biological replicates (**a, b**; means and s.e.m. of n = 3 cultures).

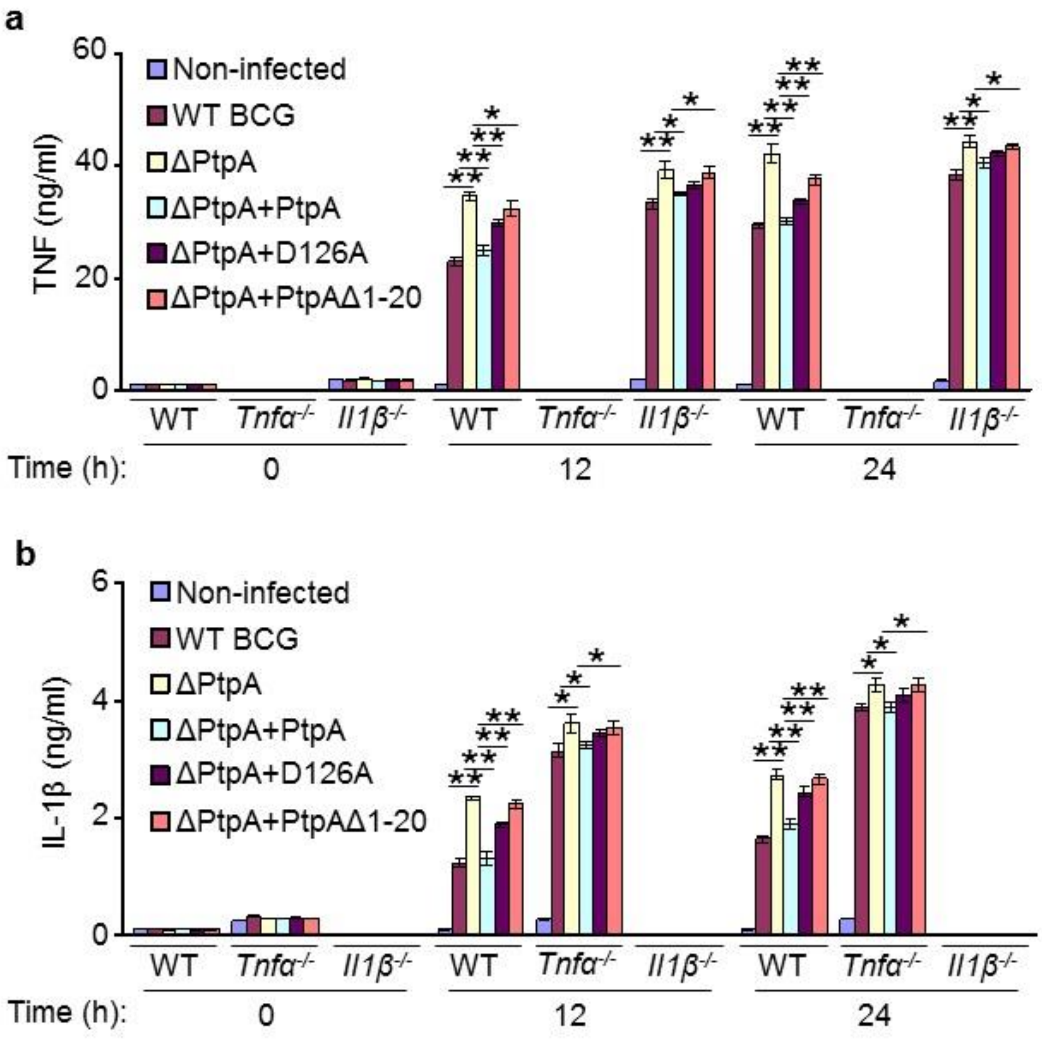


**Supplementary Figure 8: Nuclear PtpA-mediated immune suppression response is partially dependent on TNF and IL-1β. (a, b)** ELISA of TNF (**a**) and IL-1β (**b**) in the medium of the bone marrow derived macrophages (BMDMs) from WT or *Tnfα*$^{-/-}$ or *Il1β*$^{-/-}$ mice. Cells were infected with WT BCG, or BCG ΔPtpA, or BCG (ΔPtpA + PtpA), or BCG (ΔPtpA + D126A), or BCG (ΔPtpA + PtpA Δ1-20) at a MOI of 10. *$P < 0.05$ and ** $P < 0.01$ (unpaired two-tailed Student's *t* test). Data are representative of one experiment with at lease three independent biological replicates (**a, b**; means and s.e.m. of n = 3 cultures).

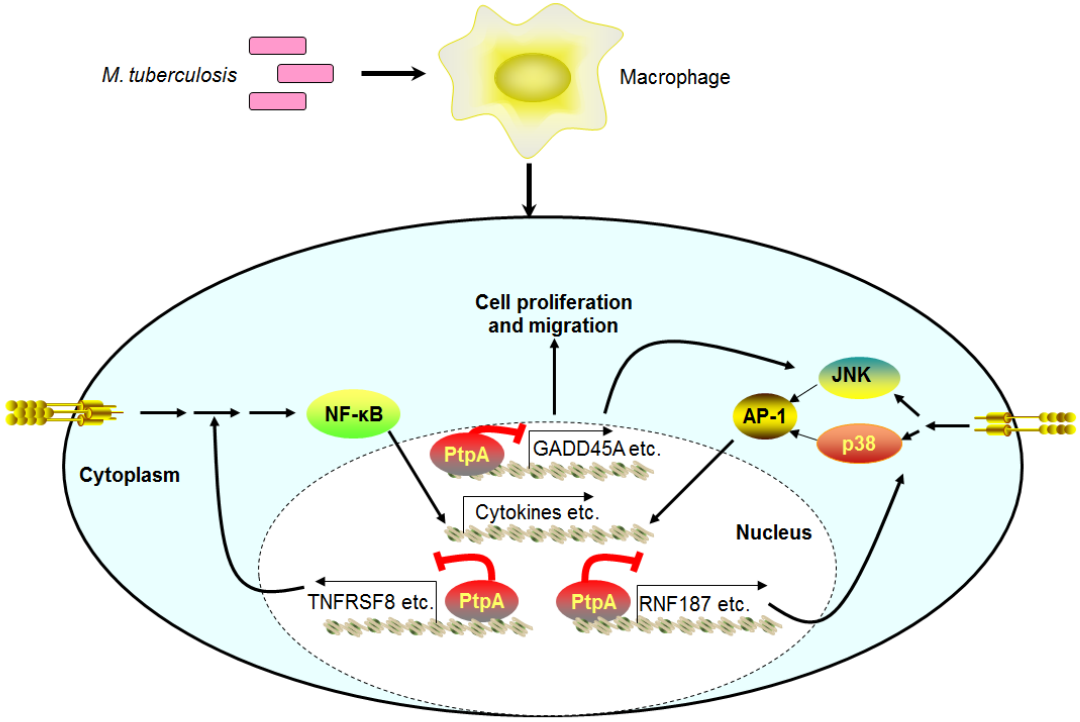


**Supplementary Figure 9: Proposed model depicting the roles of PtpA plays in the nucleus of host cells during mycobacterial infection.** PtpA inhibits the transcription of certain host genes such as *TNFRSF8* and *RNF187*, which might lead to inhibition of innate immune signaling pathways. Furthermore, PtpA promotes cell proliferation and migration of infected cells, partially through regulating the cell cycle checkpoint protein-coding gene *GADD45A*.

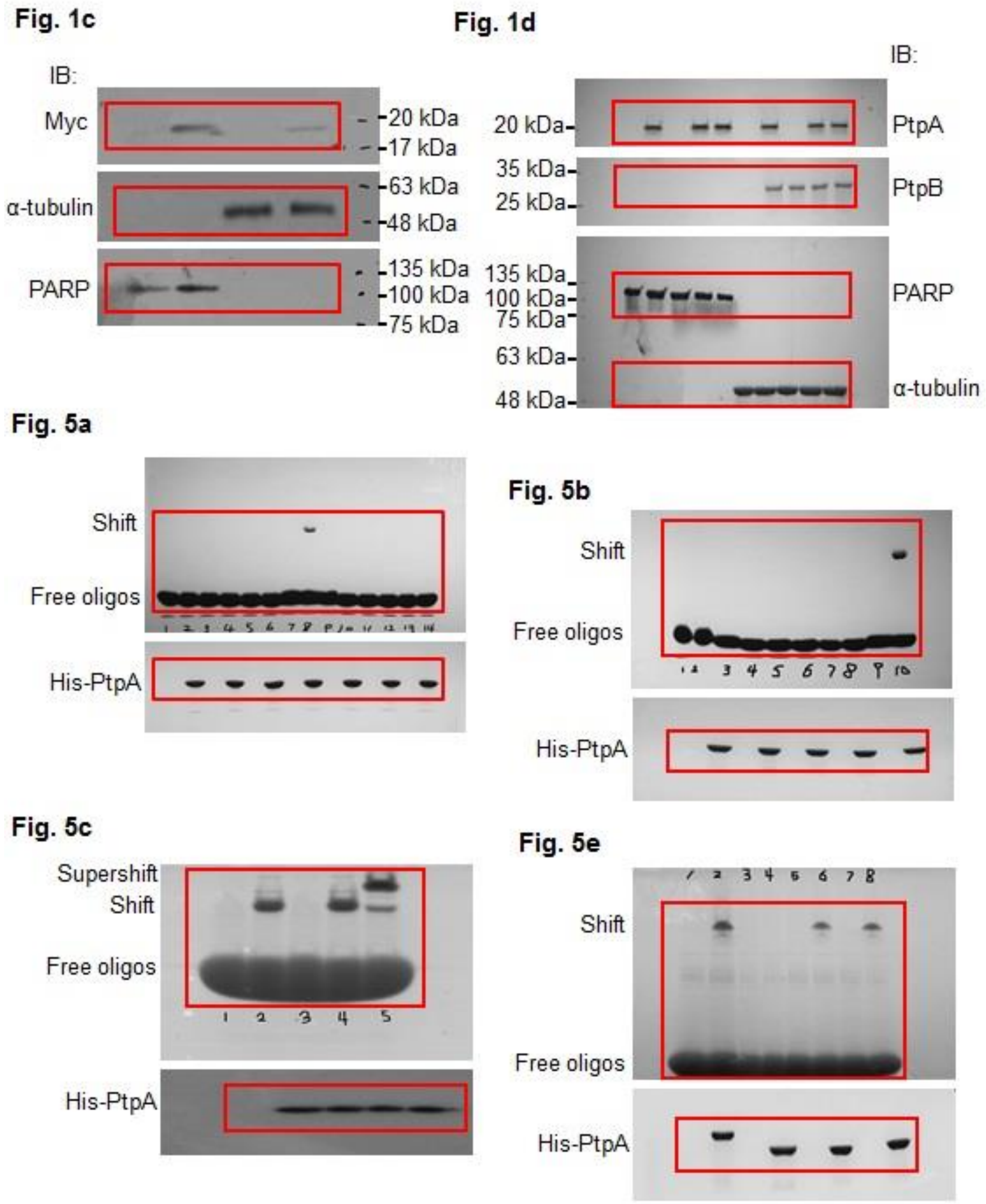


**Supplementary Figure 10: Original uncropped scans of blots for Figure 1 and 5.**

**Supplementary Table 1. Plasmids, bacterial strains and oligonucleotides used in this study.**

| Name | Description | Reference |
|---|---|---|
| **Plasmids** | | |
| pEGFP-N1 | CMV promoter, for mammalian expression, GFP tag, $Kan^R$ | Clontech |
| pEGFP-N1-PtpA | For expression of GFP-PtpA in mammalian cells | This study |
| p3xFlag-CMV14 | CMV promoter, for mammalian expression, 3xFlag tag, $Amp^R$ | Sigma |
| p3xFlag-CMV14-PtpA | For expression of Flag-PtpA in mammalian cells | This study |
| p3xFlag-CMV14-PtpA (1-20) | For expression of Flag-PtpA in mammalian cells | This study |
| pcDNA6A | T7 promoter, for mammalian expression, Myc tag, $Amp^R$ | Invitrogen |
| pcDNA6A-PtpA | For expression of Myc-PtpA in mammalian cells | This study |
| pET30a | T7 promoter, for bacterial expression, 6xHis tag, $Kan^R$ | Novagen |
| pET30a-PtpA | For expression of recombinant protein $His_6$-PtpA | This study |
| pET30a-PtpA (Δ1-20) | For expression of recombinant protein $His_6$-PtpA (Δ1-20) | This study |
| pET30a-PtpA (Δ41-49) | For expression of recombinant protein $His_6$-PtpA (Δ41-49) | This study |
| pET30a-PtpA (Δ65-75) | For expression of recombinant protein $His_6$-PtpA (Δ65-75) | This study |
| pRL-TK | Used in dual-luciferase assay for NF-κB and MAPK pathways | Feng Shao |
| pGL2-Basic | Promoterless vector for measuring the activity of promoter and enhancer sequences with a luciferase assay. | Promega |
| pGL2-Basic-GADD45A promoter | Used in dual-luciferase assay for measuring the activity of promoter | This study |
| pGL2-Basic-RNF187 promoter | Used in dual-luciferase assay for measuring the activity of promoter | This study |
| pGL2-Basic-GAS1 promoter | Used in dual-luciferase assay for measuring the activity of promoter | This study |
| pGL2-Basic-SLC35B2 promoter | Used in dual-luciferase assay for measuring the activity of promoter | This study |
| pGL2-Basic-TLK1 promoter | Used in dual-luciferase assay for measuring the activity of promoter | This study |
| pGL2-Basic-TLR7 promoter | Used in dual-luciferase assay for measuring the activity of promoter | This study |
| pGL2-Basic-MAP4K2 promoter | Used in dual-luciferase assay for measuring the activity of promoter | This study |
| pGL2-Basic-TNFRSF8 promoter | Used in dual-luciferase assay for measuring the activity of promoter | This study |
| **Strains** | | |
| *E. coli* DH5α | $F^-$ φ80*lac*ZΔM15 Δ (*lac*ZYA-*arg*F) U169 *rec*A1 *end*A1 *hsd*R17 ($r_K$-, $m_K$+) *pho*A *sup*E44 $\lambda^-$ *thi*-1 *gyr*A96 *rel*A1 | Invitrogen |
| *E.coli* BL21 (DE3) | $F^-$ *ompT hsdS*$_B$ ($r_B^-$ $m_B^-$) *gal dcm* (DE3) | Novagen |
| *M. bovis* BCG | Pasteur | ATCC 35734 |
| *M. smegmatis* | $mc^2$ 155 | ATCC 700084 |
| PtpA-*M. smegmatis* | Mtb PtpA-overexpressing *M. smegmatis* | This study |
| BCG ΔPtpA | BCG strain with deletion of PtpA | This study |

| | | |
|---|---|---|
| BCG ( Δ PtpA + PtpA) | created by introducing the integrative vector pMV306 carrying the ptpA gene and promoter into the BCG Δ PtpA strain | This study |
| BCG ( Δ PtpA + D126A) | created by introducing the integrative vector pMV306 carrying the ptpA (D126A) gene and promoter into the BCG Δ PtpA strain | This study |
| BCG ( Δ PtpA + PtpA Δ 1-20) | created by introducing the integrative vector pMV306 carrying the PtpA Δ 1-20 gene and promoter into the BCG Δ PtpA strain | This study |
| **Oligonucleotides (5’-3’)** | | |
| pEGFP-N1-PtpA-F | CCCAAGCTTGTGTCTGATCCGCTGCACGTC | This study |
| pEGFP-N1-PtpA-R | GCCTGCAGACTCGGTCCGTTCCGCGCGAGACGT | This study |
| p3xFlag-CMV14-PtpA-F | CCCAAGCTTGTGTCTGATCCGCTGCACGTC | This study |
| p3xFlag-CMV14-PtpA-R | GCTCTAGAACTCGGTCCGTTCCGCGCGAGACGT | This study |
| pcDNA6A-PtpA-F | CCCGAATTCGTGTCTGATCCGCTGCACGTC | This study |
| pcDNA6A-PtpA-R | GCCTCGAGACTCGGTCCGTTCCGCGCGAGACGT | This study |
| pET30a-PtpA-F | CCCGGATCCGTGTCTGATCCGCTGCACGTC | This study |
| pET30a-PtpA-R | GCAAGCTTCTCGGTCCGTTCCGCGCGAGACGT | This study |
| pET30a-PtpA-F (Δ1-20) | ACCTCGAGGCCGAGAAGATGTTCGCCCA | This study |
| pET30a-PtpA-R (Δ1-20) | TCTCGGCCTCGAGGTTAACGGATCCAGC | This study |
| pET30a-PtpA-F (Δ41-49) | TGCGAGTGGTAGGCAGTTGCGCCGACGA | This study |
| pET30a-PtpA-R (Δ41-49) | TGCCTACCACTCGCACCGCGTCACCCA | This study |
| pET30a-PtpA-F (Δ65-75) | GAGCCCACGTCGGCACCGAACACCTGGC | This study |
| pET30a-PtpA-R (Δ65-75) | GTGCCGACGAGGGCTCGCAACACCCCGG | This study |
| pGL2-Basic-*GADD45A* promoter-F | TCAGCTGGTGGGCGTCCAGAAGGAT | This study |
| pGL2-Basic-*GADD45A* promoter-R | ACTCACCTTTCGGTCTTCTGCTCTCC | This study |
| pGL2-Basic-*RNF187* promoter-F | TTAGCCGGGCGTCGTGGCAGGCGCC | This study |
| pGL2-Basic-*RNF187* promoter-R | CGCTGGCACAGGGCGCAGGCGGCCTC | This study |
| pGL2-Basic-*TNFRS*F8 promoter-F | CTTATGCATCCATCCATCCATCCAT | This study |
| pGL2-Basic-*TNFRSF8* promoter-R | CGTAGCGCCCCCAGGAACAGCAGTCCC | This study |
| pGL2-Basic-*GAS1* promoter-F | CGTCAGGCTCCCTAAGCCGTTCCTT | This study |
| pGL2-Basic-*GAS1* promoter-R | ACTGTCCCCCCGCGGGCCTCGCCGCC | This study |
| pGL2-Basic-*SLC35B2* promoter-F | TGGGGCAATAAACCAGCACAGTCTC | This study |
| pGL2-Basic-*SLC35B2* promoter-R | GTAAAGCGCTCACCCGGTGATGTGG | This study |
| pGL2-Basic-*TLR7* promoter-F-R | CAGGTGGACTAAGTAGATTAAAGAA | This study |
| pGL2-Basic-*TLR7* promoter-R | CAAAGATTACAGATAACACTTTTTA | This study |
| pGL2-Basic-*MAP4K2* promoter-F | AGCACAGGTCCCACCTCCAGCCCGG | This study |
| pGL2-Basic-*MAP4K2* promoter-R | TCGAAGCGGTCCCGCGGGTCCTGCAG | This study |

| | | |
|---|---|---|
| *GADD45A* promoter (-700~-550bp)-F | TCAGCTGGTGGGCGTCCAGAAGGAT | This study |
| *GADD45A* promoter (-700~-550bp)-R | GGTTTCCCACCTGCCCGGTGTAACT | This study |
| *GADD45A* promoter (-550~-400bp)-F | GTTCTGCTTTCTGTGGAAAAGATTCT | This study |
| *GADD45A* promoter (-550~-400bp)-R | AGCAGTAGTATTATTTCGGTGCCCTG | This study |
| *GADD45A* promoter (-400~-250bp)-F | AATAAGCAGCTTCGCCTAGACTTAGA | This study |
| *GADD45A* promoter (-400~-250bp)-R | GGTGGCTATTAAAATCTATTTCCAGG | This study |
| *GADD45A*promoter (-250~-100bp)-F | CCTTAAAACAAAAGACATGAAAAGAT | This study |
| *GADD45A* promoter (-250~-100bp)-R | TTATCCGGTTGGCGGGGACCATTGG | This study |
| *GADD45A* promoter (-100~+50bp)-F | GAGTGCGCGCGGGACCCGCCTTCCC | This study |
| *GADD45A* promoter (-100~+50bp)-R | ACTCACCTTTCGGTCTTCTGCTCTCC | This study |
| GADD45A-QRT-F | ATCCTGCGCGTCAGCAACCC | This study |
| GADD45A-QRT-R | TGCACTGCGTGCTGGTGACG | This study |
| RNF187-QRT-F | CGGCGCCCGCGCGCGACGGC | This study |
| RNF187-QRT-R | CCCCGAGCCGCCCGAGTGGG | This study |
| GAS1-QRT-F | TGGGCTGCACCGAGGCCCGG | This study |
| GAS1-QRT-R | GCGCTGCACGGACGAATGCC | This study |
| TNFRSF8-QRT-F | TGTTCTCGAGACGACCTCGT | This study |
| TNFRSF8-QRT-R | GAGCCAGTACGACTCTGGCA | This study |
| Gapdh-QRT-F | GGAGCGAGATCCCTCCAAAAT | This study |
| Gapdh-QRT-R | GGCTGTTGTCATACTTCTCATGG | This study |
| MAP4K2-QRT-F | CTCCAGGGAGATGTCAAACT | This study |
| MAP4K2-QRT-R | CTCCCGAGGTGGCTGCTGTG | This study |
| SLC35B2-QRT-F | CTCTGATGAGGTTCCCCTGG | This study |
| SLC35B2-QRT-R | TCTTATCTGACTTGGGGTGT | This study |
| TLR7-QRT-F | GGATGGAAAC CAGCTACTAG | This study |
| TLR7-QRT-R | CTAACAGAACTGGCCAACAT | This study |
| *Tnf*-QRT-F | GAACTGGCAGAAGAGGCACT | This study |
| *Tnf*-QRT-R | CGTGGTGGCCCCTGCCACAAG | This study |
| *Il1b*-QRT-F | TGGGATGATG ATGATAACCT | This study |
| *Il1b*-QRT-R | GGTCCGACAGCACGAGGCTT | This study |
| *Il12b*-QRT-F | ACCCTGACCATCCAAGTCAAA | This study |
| *Il12b*-QRT-R | TTGGCCTCGCATCTTAGAAAG | This study |
